# Effects of water extracts of *Artemisia annua* L. on rumen immune and antioxidative indexes, fermentation parameters and microbials diversity in lambs

**DOI:** 10.3389/fmicb.2024.1485882

**Published:** 2024-10-18

**Authors:** Gen Gang, Ruiheng Gao, Huricha Zhao, Yuanqing Xu, Yuanyuan Xing, Xiao Jin, Lei Hong, Sumei Yan, Binlin Shi

**Affiliations:** College of Animal Science, Inner Mongolia Agricultural University, Hohhot, China

**Keywords:** water extracts of *Artemisia annua* L, lamb, immune and antioxidative index, rumen fermentation, microbial diversity

## Abstract

The present study investigated the effects of water extracts of *Artemisia annua* L. (WEAA) on rumen immune and antioxidative indexes, fermentation parameters and microbial diversity in lambs. A total of 32 3-month-old Dorper × Han female lambs having comparable body weights (24±0.09 kg) were selected and were randomly assigned to four treatments, with eight repetitions for each treatment. The basal diet, consisting of 45% concentrate and 55% forage, was solely provided to the control group. For the other treatment groups, the basal diet was supplemented with WEAA at dosages of 500, 1000, and 1500 mg/kg diet, respectively. Rumen tissue samples were collected for the analysis of immune and antioxidative parameters, as well as related gene expression. Rumen fluid samples were collected to assess rumen fermentation parameters on days 30 and 60 and to evaluate the microbiota on day 60. Results showed that WEAA supplementation linearly or quadratically increased the content of sIgA, IL-4, IL-2 and the gene expression level of MyD88, IκB-α, IL-4, COX-2, iNOS in rumen tissue (*p* < 0.05), as well as the bacteria negatively associated with IL-6 (*g_ [Eubacterium]_cellulosolvens_group*). Furthermore, the addition of WEAA linearly or quadratically increased rumen T-SOD, GSH-Px (*p* < 0.05) and the gene expression level of Nrf2, SOD2, GSH-Px, HO-1 (*p* < 0.05), and decreased the rumen concentration of malondialdehyde (MDA) and gene expression level of Keap1 (*p* < 0.05), as well as the bacteria positively associated with T-AOC, T-SOD and GSH-Px (*g_Lachnospiraceae_NK3A20_group*, *g_Saccharofermentans*, *g__Marvinbryantia*, *g_unclassified_f_Eggerthellaceae*). The supplementation of WEAA caused the concentration of microprotein (MCP), total volatile fatty acids (TVFA), propionate to increase either linearly or quadratically, while reducing the concentration of NH3-N and the acetate/propionate ratio (A:P) in rumen fluid (*p* < 0.05). The addition of WEAA linearly or quadratically increased the abundance of Actinobacteriota, Cyanobacteria and Lachnospiraceae_NK3A20_group (*p* < 0.10), and *g__Lachnospiraceae_NK3A20_group*, *g_Saccharofermentans*, *g_Marvinbryantia*, g_Bifidobacterium were significantly abundant as specific microflora in the 1000 mg/kg WEAA supplementation group. In conclusion, dietary inclusion of 1000 mg/kg WEAA improved the rumen immune function, antioxidant status, rumen fermentation, and composition of rumen microbes in lambs.

## 1 Introduction

Sheep are one of the most significant livestock species worldwide, providing meat, milk, and wool. Economic growth, accompanied by an increasing demand for mutton, has driven rapid advancements in the sheep industry (Zeder, 2008). In contemporary sheep production, China’s sheep sector has transitioned from being extensive to modern and intensive. This shift has altered the living environment and natural behavioral patterns of sheep, subsequently affecting their normal metabolism and immune function. In addition, there are still various problems, for example, the collocation of nutrient substances is commonly neglected when feeding sheep, which can have adverse effects on the immunity and health of sheep. The health and productivity of lambs heavily depend on the composition of their diet. In the modern livestock production system, the research on natural plant-derived feed additives to achieve green, environmental protection, safety and extreme efficiency has become one of the focuses in the field of animal production. Natural plant feed additives, derived from natural plants, maintain the innate state and biological activity of their various components and structures. They are characterized by the absence of drug resistance, residue, and toxic side effects. Genus Artemisia plants have been extensively researched due to its abundant active components, nutritional and medicinal values.

*Artemisia annua* L., a member of the family Asteraceae (formerly Compositae), is a traditional Chinese herbal medicine recognized for its role as a source of artemisinin, and distributes throughout countries in America, Europe, and Asia (McVaugh, 1984). There has been considerable research on the *Artemisia annua* L. and its extracts owing to their significant concentration of terpenoid compounds and being abundant in polysaccharides, flavonoids and essential oils (Aftab et al., 2014; Shinyuy et al., 2023; Brown, 2010). Artemisinin, a main bioactive ingredient of *Artemisia annua* L., is known for antimalarial activity all over the world. Besides antimalarial effects, the components possess significant advantages (Elfawal et al., 2015), including antioxidant and anti-inflammatory properties, as well as antibacterial functions (El-Sawy et al., 2023; Li et al., 2015; Nair et al., 2023). Niu et al. (2020) reported that enzymatically treated *Artemisia annua* L. could increase the content of the sIgA and IgG, while decrease the inflammatory factors IL-1β, IL-6 and TNF-*α* of pigs. It has been reported that the addition of 1% *Artemisia annua* L. extract to diets can increase antioxidative enzymes activity and decrease malondialdehyde (MDA) content in serum and jejunum of geese (Cui et al., 2024). In addition, it can change the structure of the cecum microbiota. Choi et al. (2020) conducted a study in which they pretreated human hepatocarcinoma cell line (HepG2 cells) with WEAA to investigate its effects on lipid accumulation and cytotoxicity, and the findings revealed that WEAA inhibited lipid accumulation within HepG2 cells and activated antioxidant enzymes. However, the research in this area mainly focused on monogastric animals, and there were only a few studies on ruminants, especially sheep. The present study investigates the effects of WEAA on the immune and antioxidant function, fermentation parameters, and microbial diversity of the rumen in lambs, thereby providing theoretical basis for the effective use of WEAA as a feed additive to promote the rumen health of sheep. Xing et al. (2019) stated that dietary supplementation with the water extract of *Artemisia ordosica* might be effective in improving the antioxidant capacity of weanling piglets. Researchers supplemented WEAA into the diet of broilers and observed that the extract exhibited potent antioxidant capacity, as well as modulated the expression of related mRNA in intestinal mucosa through the Nrf2 signaling pathway (Guo et al., 2022). *Artemisia annua* L. and its extract are abundant in terpenoids and flavonoids, which have been shown to significantly enhance the rumen fermentation function of ruminants. In cows, *Artemisia annua* extract significantly increased TVFA, propionic acid, and butyric acid concentration in rumen, as well as significantly lower the A:P (Yu et al., 2021). To summarize, most studies on the effects of artemisia plant-derived feed additives on animals are currently focused on pigs or chickens, with few studies conducted in sheep. Therefore, based on the existing literature highlighting the significant nutritional value of *Artemisia annua* L. and the reported beneficial effects in previous studies, the current experiment was formulated to examine the effect of dietary WEAA at three varying levels on rumen immune and antioxidative indexes, fermentation parameters and microbials diversity in lambs.

## 2 Materials and methods

This animal trial was conducted at a sheep farm located in Hohhot, Inner Mongolia, China. All experimental procedures were sanctioned by the Animal Care and Use Committee of Inner Mongolia Agricultural University, with the approval code being NND20211097.

### 2.1 Animals, diets, and design

A total of 32 3-month-old Dorper × Han female lambs with similar body weight (24 ± 0.09 kg) were selected and were randomly assigned to four treatments, with each treatment having eight replicates. The control group was exclusively fed with the basal diet which consisted of 45% concentrate and 55% forage including alfalfa hay, corn straw and oat grass (Table 1). Meanwhile, the treatment groups were supplied with the basal diet augmented with WEAA at dosages of 500, 1,000, and 1,500 mg/kg diet, respectively. The feeding experiment persisted for a duration of 75 days, consisting of a 15-day adaptation phase and a 60-day formal experimental period. Lambs were fed twice daily (8:00 and 16:00). The lambs were fed with pelleted rations. Experimental diets and water were available *ad libitum*. Each lamb was housed individually in pens, and feed intake was monitored to ensure that leftover feed exceeded 5% of the total feed provided. In accordance with the Nutrient Requirements of Meat-type Sheep (NY/T 816–2021), the basal diet was formulated to cover lambs’ nutritional requirements. The ingredients and nutrient composition are shown in Table 1. The contents of dry matter (DM), crude protein (CP), neutral detergent fiber (NDF), acid detergent fiber (ADF), calcium (Ca), and phosphorus (P) in the feed were determined based on GB/T 6435–2014, GB/T 6432–2018, GB/T 20806–2006, NY/T 1459–2007, GB/T 6436–2018, and GB/T 6437–2018 (National Technical Committee of Feed Industry Standardization, 2014, 2006, 2018a, 2018b, 2018c, 2007), respectively. Rumen tissue samples were collected for the analysis of immune and antioxidative parameters, as well as related gene expression. Rumen fluid samples were collected to assess rumen fermentation parameters on days 30 and 60 and to evaluate the microbiota on day 60.

**Table 1:** The composition and nutrient levels of the basal diet (on an air-dry basis).

Ingredients	Content, %	Nutrients composition ^(2)^	Level, %, unless otherwise stated
Alfalfa hay	16.25	Digestible energy (MJ/Kg)	12.01
Corn straw	14.00	Dry matter	89.89
Oat grass	24.75	Crude protein	15.80
Corn	23.25	Neutral detergent fiber	40.80
Soybean meal	10.95	Acid detergent fiber	26.24
Wheat bran	4.25	Calcium	1.08
Corn germ meal	1.95	Phosphorus	0.40
Soybean oil	1.10		
Premix ^(1)^	0.50		
Limestone	1.10		
Calcium phosphate dibasic	0.70		
Salt	0.40		
Sodium bicarbonate	0.80		
Total	100.00		

(1) The premix provided the following nutrient content for one kilogram of diet: vitamin A, 6000 IU; vitamin D3, 2,500 IU; vita-min E, 12.5 IU; vitamin K3, 31.8 mg; vitamin B1, 0.035; vitamin B2, 8.5 mg; vitamin B6, 0.9 mg; nicotinic acid, 22 mg; D-pantothenic acid, 17 mg; vitamin B12, 0.03 mg; biotin, 0.14 mg, folic acid, 1.5 mg; Fe, 0.04 g; Cu, 0.008 g; Zn, 0.05 g; Mn, 0.03 g; I, 0.3 mg, Se, 0.3 mg; Co, 0.25 mg.

(2) Digestible energy was calculated according to the nutritional requirements of meat sheep (NY/T 816–2021), while the rest were measured values.

### 2.2 Raw materials and extraction process of WEAA

The extract used in this study was obtained from *Artemisia annua* L. collected in Hohhot, Inner Mongolia. WEAA was prepared in accordance with the method outlined by Guo et al. (2020). Briefly, the harvested above-ground parts of *Artemisia annua* L. plants were dried in the shade, and then cut into short pieces and used for the extraction of water extract of *Artemisia annua* L. (WEAA) through decoction, concentration and subsequent freeze-drying. Specifically, extraction: the entire *Artemisia annua* L. plant was cut into short segments, which added to water at a ratio of 1:25 and then incubated for 7 h at 80°C; filtration: after completion of the extraction process, a vacuum filtration was conducted, and the filtrate was collected; concentration -- utilize a rotary evaporator (RE-5298, Shanghai Yarong Biochemical Instrument Factory, Shanghai, China) to concentrate the collected filtrate at 80°C in order to lower the water content; freeze drying: pour the collected concentrated solution into a culture dish and use a freeze-drying machine (ALPHA1-2LD plus, Christ, Germany) to freeze dry until all residual water is removed, and made into a powder for later use. All extracts were stored at 4°C.

### 2.3 Sample collection and analyses

#### 2.3.1 Rumen immune and antioxidative indicators

On the 60th day of the experiment, the lambs were subjected to a 12-h fasting period and subsequently euthanized for tissue sampling. The rumen tissue was finely chopped and homogenized at a 10% (w/v) ratio in saline solution. Subsequently, it was centrifuged at 3,000 rpm for 10 min at a 4°C (Shi et al., 2022). The immune indicators, including the content of Secretory immunoglobulin A (sIgA), Immunoglobulin G (IgG), Immunoglobulin M (IgM), Interleukin (IL-)1β, IL-2, IL-4, IL-6, and Tumor necrosis factor -*α* (TNF-α), were determined by employing commercial enzyme linked immunosorbent assay (ELISA) kits in line with the manufacturer’s guidelines (Wuhan Colorful Gene Biological Technology Co., LTD, Wuhan, China). The antioxidative indicators, including the activity of superoxide dismutase (SOD), catalase (CAT), glutathione peroxidase (GSH-Px), malondialdehyde (MDA), and total antioxidant capacity (T-AOC) in rumen tissue, were spectrophotometrically quantified by employing commercially accessible kits in alignment with the manufacturer’s guidelines from Jiancheng Bioengineering Institute, located in Nanjing, China.

#### 2.3.2 Rumen mRNA expression

The total RNA from rumen tissue samples was acquired by employing the Trizol™ extraction method in accordance with the manufacturer’s instructions (Accurate Biotechnology Co. Ltd., Hunan, China). The total RNA samples were quantitatively and qualitatively evaluated with a spectrophotometer (Pultton P200 CM, San Jose, CA, USA) to ascertain the absorbance ratio at 260 and 280 nm. Then, the total RNA was processed with DNaseI (TaKaRa Biotechnology Co. Ltd., Dalian, China) to eliminate any genomic DNA contamination. Using the TB^®^ Green qPCR method along with a Prime Script RT™ Master Mix kit (TaKaRa Biotechnology Co. Ltd., Dalian, China) on LifeECO (Bori Technology Co., Ltd. Hangzhou, China), total RNA was converted to cDNA. The reactions were incubated at 37°C for 15 min, followed by 5 s at 85°C. The target genes include Toll-like receptor 4 (*TLR4*), MYD88 innate immune signal transduction adaptor (*MyD88*), inhibitor of nuclear factor kappa B kinase subunit beta (*IKKβ*), NFKB inhibitor alpha (*IκB-α*), nuclear factor kappa B subunit 1 (*NFκBp50*), RELA proto-oncogene (*NFκBp65*), interleukin (IL-) 1β, *IL-4*, tumor necrosis factor -α (*TNF-α*), nitric oxide synthase 2 (*iNOS*), cyclooxygenase-2 (*COX-2*), nuclear factor erythroid 2-related factor 2 (*Nrf2*), kelch-like ECH associated protein 1 (*Keap1*), superoxide dismutase 1 (*SOD1*), superoxide dismutase 2 (SOD2), catalase (CAT), glutathione peroxidase (*GSH-Px*), heme oxygenase 1 (*HO-1*), and NAD(P)H quinone dehydrogenase 1 (*NQO1*). Their primer sequences annealing temperature and the expected PCR product length for various genes are tabulated in Table 2. Quantitative real-time PCR (qRT-PCR) was performed using the Quant Studio R5 Realtime PCR Design & Analysis system (Light Cycler® 480II, Roche Diagnostics, USA) and the TB^®^ Premix Ex Taq™ Kit (TaKaRa Biotechnology Co. Ltd., Dalian, China) in accordance with the directions of the manufacturer. All samples were processed in duplicate within a 10 μL reaction mixture. This mixture comprised 5 μL of TB green mix, 0.8 μL of each forward primer (with a concentration of 0.4 μM) and reverse primer (also at 0.4 μM), 1 μL of cDNA, and 3.2 μL of nuclease-free water. Subsequently, melt curve analysis was carried out to confirm the specificity of the PCR-amplified product. The mRNA expression of each gene was normalized with respect to that of beta-actin *(β-actin)* and glyceraldehyde-3-phosphate dehydrogenase *(GAPDH)*. The relative fold difference in the mRNA expression levels was analyzed according to the 2^−△△CT^ method (Livak and Schmittgen, 2001).

**Table 2:** Primer sequences and parameter.

Gene ^(1)^	Sequence (5′- > 3′) ^(2)^	GenBank No.	Length/bp
β-actin	F- ACAATGTGGCCGAGGACTTT	NM_001009784.3	278
	R- GCCGTGATGGCTGACCATTC		
GAPDH	F- TTATGACCACTGTCCACGCC	NM_001190390.1	216
	R- TCAGATCCACAACGGACACG		
Nrf2	F- TGTGGAGGAGTTCAACGAGC	XM_004004557.1	88
	R- CGCCGCCATCTTGTTCTTG		
Keap1	F- TTCAACAGCGAAAGTCAGGC	XM_027969637.2	157
	R- TGCGTAGCCTCCGATACTCT		
SOD1	F- GGAGACCTGGGCAATGTGAA	NM_001145185	182
	R- CCTCCAGCGTTTCCAGTCTT		
SOD2	F- AAACCGTCAGCCTTACACC	NM_001280703.1	116
	R- ACAAGCCACGCTCAGAAAC		
CAT	F- GAGCCCACCTGCAAAGTTCT	XM_004016396	148
	R- CTCCTACTGGATTACCGGCG		
GSH-Px	F- TGGTCGTACTCGGCTTCCC	XM_004018462.1	163
	R- AGCGGATGCGCCTTCTCG		
HO-1	F- CGATGGGTCCTCACACTCAG	XM_027967703.2	74
	R- CACACTCGCATTCACATGGC		
NQO1	F- CTCTGGCCAATTCAGAGTGG	XM_004015102.5	296
	R- TCCATTGGGATGGACTTGCC		
TLR4	F- CCTTGCGTACAGGTTGTTCC	NM_001135930.1	99
	R- GTCCAGCATCTCGGTTGACA		
MyD88	F- ATTGAGAAGAGGTGCCGTCG	NM_001166183.1	189
	R- ACAGACAGTGATGAAGCGCA		
IKKβ	F- GCCGCCCATTACAAGCTGAA	XM_042241396.1	165
	R- CTGGAAGAACGGGAGGTTCC		
IκB-*α*	F- TCACCTACCAGGGCTACTCC	NM_001166184.1	153
	R- CTGTGAACTCTGATTCGGTGTC		
NF-κBp50	F- GATGCCACTGCCAACAGAGA	XM_042251202.1	191
	R- GCGTCTGTCATTCGTGCTTC		
NF-κBp65	F- CTCCTCTCGGGGGATGAAGA	XM_027959295.2	123
	R- ATCCCTTGCTAACCCACTGC		
IL-1β	F- CTGTGGCCTTGGGTATCAGG	NM_001009465.2	251
	R- GCCACCTCTAAAACGTCCCA		
IL-4	F- GCTGAACATCCTCACATCGAG	AF1721681	87
	R- TTCTCAGTTGCGTTCTTTGG		
TNF-α	F- AGTCTGGGCAGGTCTACTTTG	NM_001024860	127
	R- GGTAACTGAGGTGGGAGAGG		
iNOS	F- AGACTGAGCCTCTCTAGCCC	XM_042255454.1	96
	R- GGAACCGTCTATAGCTGCCC		
COX-2	F- ACTTTCACGACCACACATTA	NC_001941.1	501
	R- GACGAGTTGACATAAGGGTT		

(1) β-actin = beta-actin; GAPDH = Glyceraldehyde-3-phosphate dehydrogenase; Nrf2 = Nuclear factor erythroid 2-related factor 2; Keap1 = kelch like ECH associated protein 1 (KEAP1); SOD1 = Superoxide dismutase 1; SOD2 = Superoxide dismutase 2; CAT = Catalase; GSH-Px = Glutathione peroxidase; HO-1 = Heme oxygenase 1; NQO1 = NAD(P)H quinone dehydrogenase 1; TLR4 = Toll like receptor 4; MyD88 = MYD88 innate immune signal transduction adaptor; IKKβ = Inhibitor of nuclear factor kappa B kinase subunit beta (IKBKB); IκB-α = NFKB inhibitor alpha (NFKBIA); NFκBp50 = Nuclear factor kappa B subunit 1 (NFKB1); NFκBp65 = RELA proto-oncogene; IL-1beta = Interleukin-1β; IL-2 = Interleukin-2; IL-4 = Interleukin-4; IL-6 = Interleukin-6; TNF-α = Tumor necrosis factor -α; iNOS = Nitric oxide synthase 2 (NOS2); COX2 = Cytochrome c oxidase subunit II.

(2) F: forward primer; R: reverse primer.

#### 2.3.3 Rumen fermentation index measurement

Rumen fluid samples (approximately 50 mL) were collected from each animal using a tube-type nasogastric sampler on the 30th day of the experimental period. All collections were executed prior to the morning feeding (at 08:00). On day 60 of the experiment period (after slaughter), rumen fluid was directly collected. The rumen fluid samples that were gathered were filtered by means of four layers of absorbent gauze for the elimination of particulate matter, and the pH value was determined subsequent to filtration with a pH meter; a sum of 1 mL of metaphosphoric acid (25 g/100 mL) was incorporated into 4 mL of rumen fluid and blended for VFA assessment using gas chromatographic (Agilent 7890A, Palo Alto, CA, USA) by Li et al. (2023); 4.5 mL of 0.2 M hydrochloric acid was put into 0.5 mL of rumen fluid and mixed to gauge NH_3_-N using colorimetric analysis by Miguel et al. (2019); the remaining rumen fluid was employed to determine microbial protein (MCP) using Coomassie brilliant blue analysis by Chanjula and Cherdthong (2018).

#### 2.3.4 Analysis of microbial community in ruminal fluid

On day 60 of the experiment period (after slaughter), ruminal fluid was collected and promptly frozen in liquid nitrogen before being kept at −80°C. Subsequently, the specimens were dispatched to Shanghai Majorbio Bio-Pharm Technology Co., Ltd. for analysis of ruminal microbial community diversity using 16SrRNA gene sequencing. The entire DNA of the microbial community from the ruminal liquid was extracted in accordance with the manufacturer’s directions using the E.Z.N.A.^®^ soil DNA Kit (Omega Bio- Tek, Norcross, GA, U.S.). DNA quality was confirmed through a 1% agarose gel electrophoresis, and the DNA concentration and purity were checked with a NanoDrop2000. For the analysis of microbial diversity, PCR amplification of the V3-V4 variable regions of the 16SrRNA gene was executed with universal primers: the forward primer 338F having a sequence of 5′-ACTCCTACGGGAGGCAGCAG-3′ and the reverse primer 806R with a sequence of 5’-GGACTACHVGGGTWTCTAAT-3′. As part of PCR analysis process, the PCR products were mixed and recovered on 2% agarose gel, purified by the AxyPrep DNA Gel Extraction Kit (Axygen Biosciences, Union City, CA, USA), and homogenized and quantified by 2% agarose gel electrophoresis and Quantus™ Fluorometer (Promega, USA). Sequencing library was prepared using NEXTFLEX Rapid DNA-Seq Kit, and the qualified library was sequenced with Illumina Miseq PE300 platform (Illumina, San Diego, CA, USA) (Guo et al., 2024).

The quality control of the initial sequences was carried out with the utilization of Fastp software and Flash software to merge the eligible raw data from the preceding stage, thereby attaining the complete paired end sequence. The operational taxonomic unit (OTU) clustering was conducted at a 97% sequence resemblance through the Uparse software. Through OTU clustering, the OTU representative sequences were acquired. Subsequently, the elimination of chimeras was implemented for non-repetitive sequences to create an OTU table. Finally, all the representative sequences were compared with the Silva 16S rRNA database by using the RDP classifier to obtain the OTU annotation information, and the threshold was set at 0.7 (Ren et al., 2022).

### 2.4 Statistical analysis

All data were initially processed with Microsoft Excel 2021 and subsequently analyzed through a regression analysis using the SAS Version 9.2 (2008, SAS Institute, Cary, NC, USA), namely, all the data were analyzed to assess the linear and quadratic impacts on the diverse indexes responding to increasing dietary WEAA levels using the SAS, thereby determining the dose-dependent effects of each index on WEAA. The outcomes were presented as means along with the standard error of the mean (SEM), and the probability value of *p* ≤ 0.05 were considered as the criterion for statistical significance, while a probability value of 0.05 < *p* < 0.10 was considered to indicate a tendency.

## 3 Result

### 3.1 Rumen immune indexes and relevant mRNA expression

The impact of WEAA on immune indexes in the rumen of lambs is presented in Table 3. As the supplementation of WEAA rises, the sIgA level shows a linear or quadratic increase (*p* < 0.05). Supplementation of WEAA did not exert a significant impact on the contents of IgG, IgM, IL-1β, IL-6, and TNF-*α* in the rumen tissue (*p* > 0.10). There was a quadratic increase in the IL-2 content of the rumen tissue (*p* < 0.05), with highest value for 1,000 mg/kg WEAA group. In addition, the IL-4 content in rumen tissue increased linearly or quadratically (*p* < 0.05).

**Table 3:** Effects of WEAA on immune indexes in rumen tissue of lambs.

Item ^(1)^	WEAA mg/kg ^(2)^				SEM ^(3)^	*P*-Value ^(4)^	
	0	500	1,000	1,500		Linear	Quadratic
sIgA, μg/ mg prot.	3.18	3.73	3.97	3.64	0.08	0.024	0.001
IgG, μg/ mg prot.	4.13	3.96	4.17	4.14	0.27	0.881	0.971
IgM, μg/ mg prot.	3.61	3.75	3.59	3.45	0.25	0.624	0.788
IL-1β, pg./mg prot.	58.72	57.65	61.21	56.11	3.41	0.724	0.714
IL-2, pg./ mg prot.	44.15	48.98	51.54	50.68	3.18	0.443	0.033
IL-4, pg./ mg prot.	21.12	26.14	28.62	27.21	1.68	<0.001	<0.001
IL-6, pg./ mg prot.	25.68	26.94	27.33	24.75	1.83	0.715	0.389
TNF-α, pg./mg prot.	61.58	63.78	68.91	64.41	4.07	0.198	0.155

(1) sIgA = Secretory immunoglobulin A; IgM = Immunoglobulin M; IgG = Immunoglobulin G; IL-1beta = Interleukin-1β; IL-2 = Interleukin-2; IL-4 = Interleukin-4; IL-6 = Interleukin-6; TNF-α = Tumor necrosis factor -α.

(2) Values were expressed as the mean of 8 lambs in each group and the same below. WEAA: Water Extracts of *Artemisia annua* L.

(3) SEM = Standard error of the mean.

(4) Dose-dependent effects of WEAA. The probability value of *p* ≤ 0.05 was considered to be statistically significant, whereas the probability value of 0.05 < *p* < 0.10 was considered as a tendency.

In Table 4, relative gene expression of immune indexes was shown. With the increase of WEAA supplementation, *MyD88* gene expression of rumen tissue was quadratically promoted (*p* < 0.05). Supplementation of WEAA did not exert a significant impact on the gene expression of *TLR4*, *IKKβ*, *NFκBp50*, *NFκBp65*, *IL-1β*, and *TNF-α* in rumen tissue (*p* > 0.10). Supplementing WEAA demonstrated a notable linear or quadratic effect in enhancing the expression levels of the genes *IκB-α* and *IL-4* (*p* < 0.05), which was almost similar to that of IL-4 levels. In addition, the gene expression level of *COX-2* and *iNOS* showed linear or quadratic decrease (*p* < 0.05).

**Table 4:** Effects of WEAA on expression of immune-related genes in rumen tissue of lambs.

Item ^(1)^	WEAA mg/kg ^(2)^				SEM ^(3)^	*P*-Value ^(4)^	
	0	500	1,000	1,500		Linear	Quadratic
TLR4	1.00	1.03	1.10	0.91	0.09	0.740	0.559
MyD88	1.00	1.42	1.45	1.12	0.11	0.264	0.008
IKKβ	1.00	1.01	1.09	1.08	0.15	0.495	0.795
IκB-α	1.00	1.52	1.89	1.31	0.12	0.029	<0.001
NFκBp50	1.00	0.99	1.08	0.94	0.08	0.775	0.742
NFκBp65	1.00	1.22	1.43	1.14	0.07	0.395	0.192
IL-1β	1.00	1.11	1.16	1.07	0.05	0.597	0.587
IL-4	1.00	1.72	2.03	1.54	0.11	0.046	0.002
TNF-α	1.00	0.92	1.02	1.00	0.10	0.914	0.984
COX-2	1.00	0.42	0.48	0.38	0.07	<0.001	<0.001
iNOS	1.00	0.48	0.34	0.42	0.06	<0.001	<0.001

(1) TLR4 = Toll like receptor 4; MyD88 = MYD88 innate immune signal transduction adaptor; IKKβ = Inhibitor of nuclear factor kappa B kinase subunit beta (IKBKB); IκB-α = NFKB inhibitor alpha (NFKBIA); NFκBp50 = Nuclear factor kappa B subunit 1 (NFKB1); NFκBp65 = RELA proto-oncogene; IL-1beta = Interleukin-1β; IL-4 = Interleukin-4; TNF-α = Tumor necrosis factor -α; iNOS = Nitric oxide synthase 2 (NOS2); COX2 = Cytochrome c oxidase subunit II.

(2) Values were expressed as the mean of 8 lambs in each group and the same below. WEAA: Water Extracts of *Artemisia annua* L.

(3) SEM = Standard error of the mean.

(4) Dose-dependent effects of WEAA. The probability value of *p* ≤ 0.05 was considered to be statistically significant, whereas the probability value of 0.05 < *p* < 0.10 was considered as a tendency.

### 3.2 Rumen antioxidative indexes and relevant mRNA expression

The effect of WEAA on the antioxidative index in the rumen tissue of lambs is depicted in Table 5. The supplementation of WEAA did not have significant influences on the T-AOC and CAT activity in rumen tissue (*p* > 0.10). With increased WEAA supplementation, T-SOD concentration in rumen tissue exhibited a quadratic increase (*p* < 0.05), and the activity of GSH-Px increased linearly or quadratically (*p* < 0.05), in addition, rumen MDA concentration decline linearly or quadratically (*p* < 0.05).

**Table 5:** Effects of WEAA on antioxidative indexes in rumen tissue of lambs.

Item ^(1)^	WEAA mg/kg ^(2)^				SEM ^(3)^	*P*-Value ^(4)^	
	0	500	1,000	1,500		Linear	Quadratic
T-AOC, μmol/ mg prot.	0.10	0.08	0.10	0.10	0.01	0.362	0.435
T-SOD, U/ mg prot.	172.53	173.58	183.55	168.86	17.84	0.542	0.049
GSH-Px, U/ mg prot.	23.95	23.38	27.04	28.28	2.56	<0.001	0.001
CAT, U/ mg prot.	2.45	2.67	2.56	2.50	0.18	0.966	0.684
MDA, nmol/ mg prot.	0.34	0.31	0.31	0.25	0.02	0.008	0.024

(1) T-AOC = Total antioxidant capacity; T-SOD = Total superoxide dismutase; GSH-Px = Glutathione peroxidase; CAT = Catalase; MDA = Malondialdehyde.

(2) Values were expressed as the mean of 8 lambs in each group and the same below. WEAA: Water Extracts of *Artemisia annua* L.

(3) SEM = Standard error of the mean.

(4) Dose-dependent effects of WEAA. The probability value of *p* ≤ 0.05 was considered to be statistically significant, whereas the probability value of 0.05 < *p* < 0.10 was considered as a tendency.

According to the data presented in Table 6, with increased WEAA supplementation, *Nrf2, HO-1and GSH-Px* gene expression increased linearly or quadratically (*p* < 0.05), while the expression of the *Keap1* gene exhibited a linear or quadratic decrease (*p* < 0.05). Moreover, *SOD2* gene expression tended to increase linearly or quadratically (*p* < 0.10) and NQO1 gene expression exhibited a linear increase (*p* < 0.05). However, Supplementation of WEAA did not exert a significant impact on the *SOD1* and *CAT* gene expression in rumen tissue (*p* > 0.10).

**Table 6:** Effects of WEAA on expression of antioxidant-related genes in rumen tissue of lambs.

Item ^(1)^	WEAA mg/kg ^(2)^				SEM ^(3)^	*P*-Value ^(4)^	
	0	500	1,000	1,500		Linear	Quadratic
Nrf2	1.00	1.41	1.48	1.39	0.15	0.003	<0.001
Keap1	1.00	0.65	0.43	0.42	0.06	<0.001	<0.001
SOD1	1.00	1.05	1.02	0.90	0.06	0.883	0.926
SOD2	1.00	1.29	1.63	1.42	0.10	0.056	0.074
CAT	1.00	0.99	0.94	0.93	0.08	0.734	0.945
GSH-Px	1.00	1.32	1.45	1.29	0.14	<0.001	<0.001
HO-1	1.00	1.26	1.39	1.38	0.16	0.010	0.017
NQO1	1.00	1.07	0.97	0.90	0.09	0.010	0.613

(1) Nrf2 = Nuclear factor erythroid 2-related factor 2; Keap1 = kelch like ECH associated protein 1 (KEAP1); SOD1 = Superoxide dismutase 1; SOD2 = Superoxide dismutase 2; CAT = Catalase; GSH-Px = Glutathione peroxidase; HO-1 = Heme oxygenase 1; NQO1 = NAD(P)H quinone dehydrogenase 1.

(2) Values were expressed as the mean of 8 lambs in each group and the same below. WEAA: Water Extracts of *Artemisia annua* L.

(3) SEM = Standard error of the mean.

(4) Dose-dependent effects of WEAA. The probability value of *p* ≤ 0.05 was considered to be statistically significant, whereas the probability value of 0.05 < *p* < 0.10 was considered as a tendency.

### 3.3 Rumen fermentation characteristics

As shown in Table 7, the ruminal pH levels varied between 6.7 and 7.2, showing no significant changes due to WEAA treatment (*p* > 0.10). On day 30, with increased WEAA Supplementation, the NH_3_-N concentration had a linear or quadratic (*p* < 0.05) decrease, and the MCP concentration showed a linear or quadratic increase (*p* < 0.05). On day 60, the NH_3_-N concentration showed linear or quadratic decrease (*p* < 0.05), and the MCP concentration showed significant linear or quadratic increase (*p* < 0.05).

**Table 7:** Effects of WEAA ruminal fermentation parameters of lambs.

Item ^(1)^	WEAA mg/kg ^(2)^				SEM ^(3)^	*P*-Value ^(4)^	
	0	500	1,000	1,500		Linear	Quadratic
30 d							
pH	7.02	7.20	7.05	7.16	0.04	0.482	0.717
NH_3_-N, mg/dL	29.28	20.91	23.42	22.51	0.82	0.013	0.002
MCP, g/dL	19.26	21.48	29.73	28.44	1.70	0.016	0.050
60 d							
pH	6.87	6.86	6.78	6.79	0.04	0.396	0.699
NH_3_-N, mg/dL	22.74	20.60	17.25	18.98	0.69	0.015	0.017
MCP, g/dL	22.88	24.25	31.76	28.90	1.04	0.004	0.009

(1) pH = Potential of hydrogen; NH_3_-N = Ammonia nitrogen; MCP = Microprotein.

(2) Values were expressed as the mean of 8 lambs in each group and the same below. WEAA: Water Extracts of *Artemisia annua* L.

(3) SEM = Standard error of the mean.

(4) Dose-dependent effects of WEAA. The probability value of *p* ≤ 0.05 was considered to be statistically significant, whereas the probability value of 0.05 < *p* < 0.10 was considered as a tendency.

As shown in Table 8, on day 30, with increased WEAA supplementation, the concentration of TVFA, propionic acid, isobutyric acid, and isovaleric acid showed a linear or quadratic increase (*p* < 0.05). Supplementation of WEAA did not exert a significant impact on the acetic acid, butyric acid and valeric acid concentration in rumen (*p* > 0.10). Additionally, the A:P decreased either linearly or quadratically (*p* < 0.05). On day 60, the TVFA concentration showed a quadratic increase (*p* < 0.05), and the propionic acid concentration showed a linear or quadratic increase (*p* < 0.05). Moreover, the butyric acid concentration tended to increase linearly (*p* < 0.10). The A:P decreased linearly or quadratically (*p* < 0.05). However, supplementation of WEAA did not exert a significant impact on the concentration of acetic acid, isobutyric acid, valeric acid, and isovaleric acid in rumen (*p* > 0.10).

**Table 8:** Effects of WEAA ruminal VFA of lambs.

Item ^(1)^	WEAA mg/kg ^(2)^				SEM ^(3)^	*P*-Value ^(4)^	
	0	500	1,000	1,500		Linear	Quadratic
30 d							
TVFA, mmol/L	33.83	35.37	42.17	38.29	4.26	0.031	0.040
Acetic acid, mmol/L	22.06	21.71	23.57	22.30	0.85	0.750	0.927
Propionic acid, mmol/L	5.22	6.64	10.51	8.73	0.73	0.001	0.001
Butyric acid, mmol/L	4.93	5.11	5.90	5.04	0.30	0.674	0.633
Isobutyric acid, mmol/L	0.46	0.58	0.66	0.68	0.05	<0.0001	<0.0001
Valeric acid, mmol/L	0.56	0.56	0.65	0.62	0.05	0.224	0.464
Isovaleric acid, mmol/L	0.60	0.79	0.89	0.92	0.08	0.001	0.001
A:P	4.28	3.31	3.19	3.42	0.32	0.010	0.001
60 d							
TVFA, mmol/L	82.78	89.60	94.33	86.88	8.61	0.13	0.003
Acetic acid, mmol/L	55.28	55.33	56.44	53.68	0.55	0.463	0.347
Propionic acid, mmol/L	12.58	16.83	18.92	16.27	1.73	0.033	0.002
Butyric acid, mmol/L	12.24	14.42	15.83	13.95	1.70	0.098	0.259
Isobutyric acid, mmol/L	0.62	0.65	0.71	0.66	0.03	0.482	0.613
Valeric acid, mmol/L	1.33	1.46	1.67	1.50	0.17	0.221	0.255
Isovaleric acid, mmol/L	0.72	0.90	0.74	0.82	0.05	0.783	0.817
A:P	4.49	3.75	3.27	3.35	0.35	0.003	0.005

(1) TVFA = Total volatile fatty acid; A:P = Acetate/propionate ratio.

(2) Values were expressed as the mean of 8 lambs in each group and the same below. WEAA: Water Extracts of *Artemisia annua* L.

(3) SEM = Standard error of the mean.

(4) Dose-dependent effects of WEAA. The probability value of *p* ≤ 0.05 was considered to be statistically significant, whereas the probability value of 0.05 < *p* < 0.10 was considered as a tendency.

### 3.4 Rumen microbiota diversity

#### 3.4.1 OTU cluster analysis

Analysis of rumen microbiome diversity from 32 samples resulted in a total of 2,173,498 optimized sequences, the number of optimized sequence bases was 908,855,774 bases (bp), and the average length of the quality sequences amounted to 418 bp, and the sequences of high-quality were effectively grouped and examined into OTUs that are characterized by a similarity of 97%, and a total of 3,512 OTUs being acquired. These OTUs pertained to 18 phyla, 33 classes, 69 orders, 119 families, 258 genera, and 582 species. In the subsequent analysis, in order to reduce statistical errors, the total count of sequences in every sample was reduced to 45,134.

#### 3.4.2 Alpha diversity analysis

##### 3.4.2.1 Rarefaction curve

**Figure 1:**
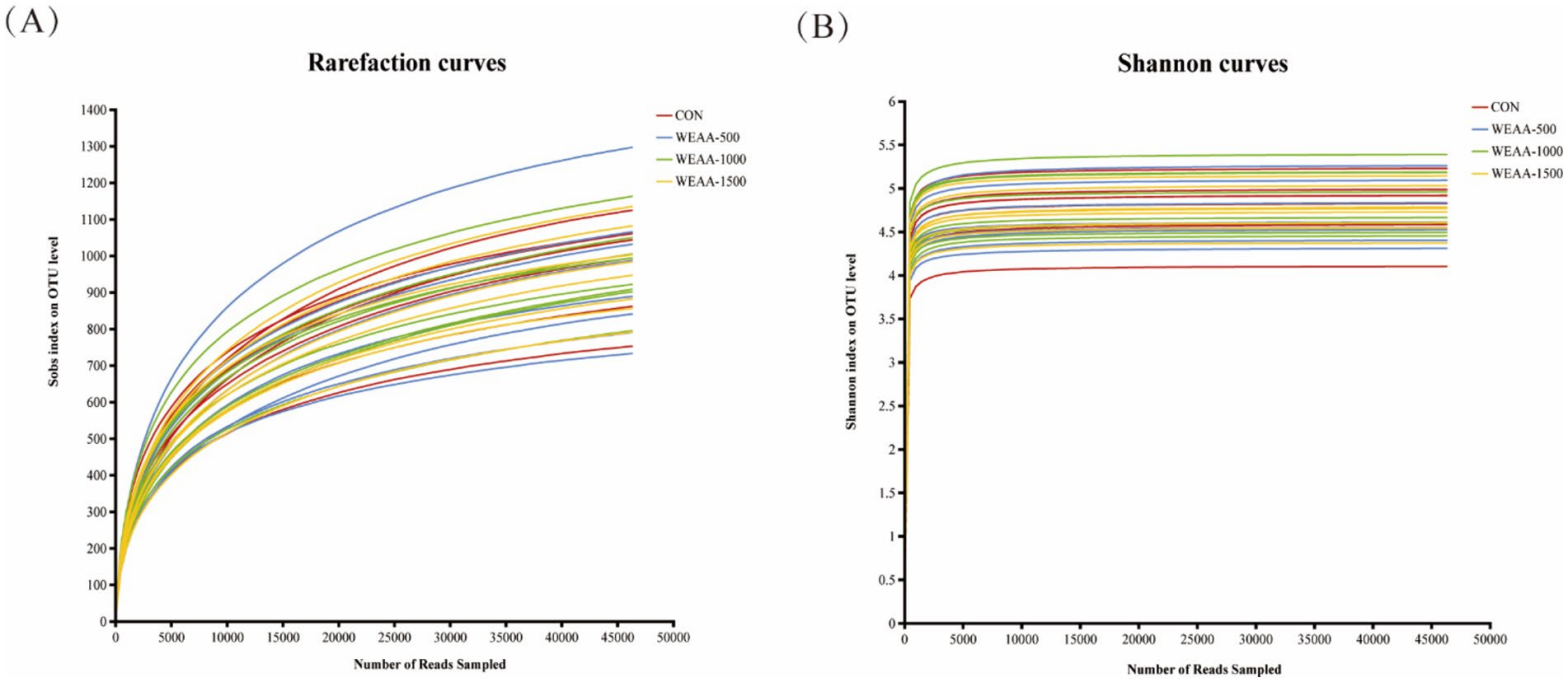
Effects of WEAA on ruminal fluid microbial diversity. **(A)** The rarefaction curves. **(B)** The Shannon curves. Values were expressed as the mean of 8 lambs in each group and the same below. WEAA: Water Extracts of *Artemisia annua* L. CON: control; WEAA-500: 500 mg/kg WEAA; WEAA-1000: 1000 mg/kg WEAA; WEAA-1500: 1500 mg/kg WEAA.

The amount of sequencing data is used as abscissa, and the number of species observed is used as ordinate. Rarefaction curves (Figure 1A) showed that when the sequencing volume was within 15,000, the curve increased significantly and the sample volume was relatively few. With the progress of sampling, the amount of sequencing data increased, the OTUs level gradually increased, and the number of observed species increased, until finally they tended to be flat, and the number of newly observed species was small. It was demonstrated that rarefaction curves tended to move towards saturation, indicating that the amount of sequencing data was adequate and that the vast majority of microorganisms in each sample had been encompassed, thereby fulfilling the requirements for rumen microbiota diversity analysis. Combined with Shannon curves (Figure 1B), our sequencing depth was sufficient to contain most microbial information and sample quality meets sequencing and analysis requirements.

##### 3.4.2.2 Alpha diversity analysis

Ace, Chao1, Shannon, Simpson, and Coverage indices were obtained by analysis of the Alpha diversity index, as shown in Table 9. All samples had an OTU Good’s coverage index value of more than 99% (0.9959), indicating that the library constructed in this study could effectively reflect the abundance and diversity of the microbial community of the samples. The inclusion of WEAA in the diet did not lead to a substantial dissimilarity in the Ace, Chao1, Shannon, and Simpson indices between the control group and the WEAA supplementation group (*p* > 0.05). As a result, WEAA did not exert adverse effects on the rumen microbial richness (as signified by the Ace and Chao1 indices) and diversity (as depicted by the Shannon diversity index and Simpson diversity index) of lambs.

**Table 9:** Effects of WEAA on the rumen microbial alpha diversity of lambs.

Item	WEAA mg/kg ^(1)^				SEM ^(2)^	*P*-Value ^(3)^	
	0	500	1,000	1,500		Linear	Quadratic
Ace	1136.40	1097.27	1134.58	1102.81	25.05	0.782	0.961
Chao	1112.54	1086.30	1122.25	1083.64	24.23	0.819	0.967
Shannon	4.77	4.69	4.86	4.74	0.06	0.846	0.961
Simpson	0.03	0.03	0.03	0.03	0.01	0.599	0.826

(1) Values were expressed as the mean of 8 lambs in each group and the same below. WEAA: Water Extracts of *Artemisia annua* L.

(2) SEM = Standard error of the mean.

(3) Dose-dependent effects of WEAA. The probability value of *p* ≤ 0.05 was considered to be statistically significant, whereas the probability value of 0.05 < *p* < 0.10 was considered as a tendency.

#### 3.4.3 Beta diversity indices

**Figure 2:**
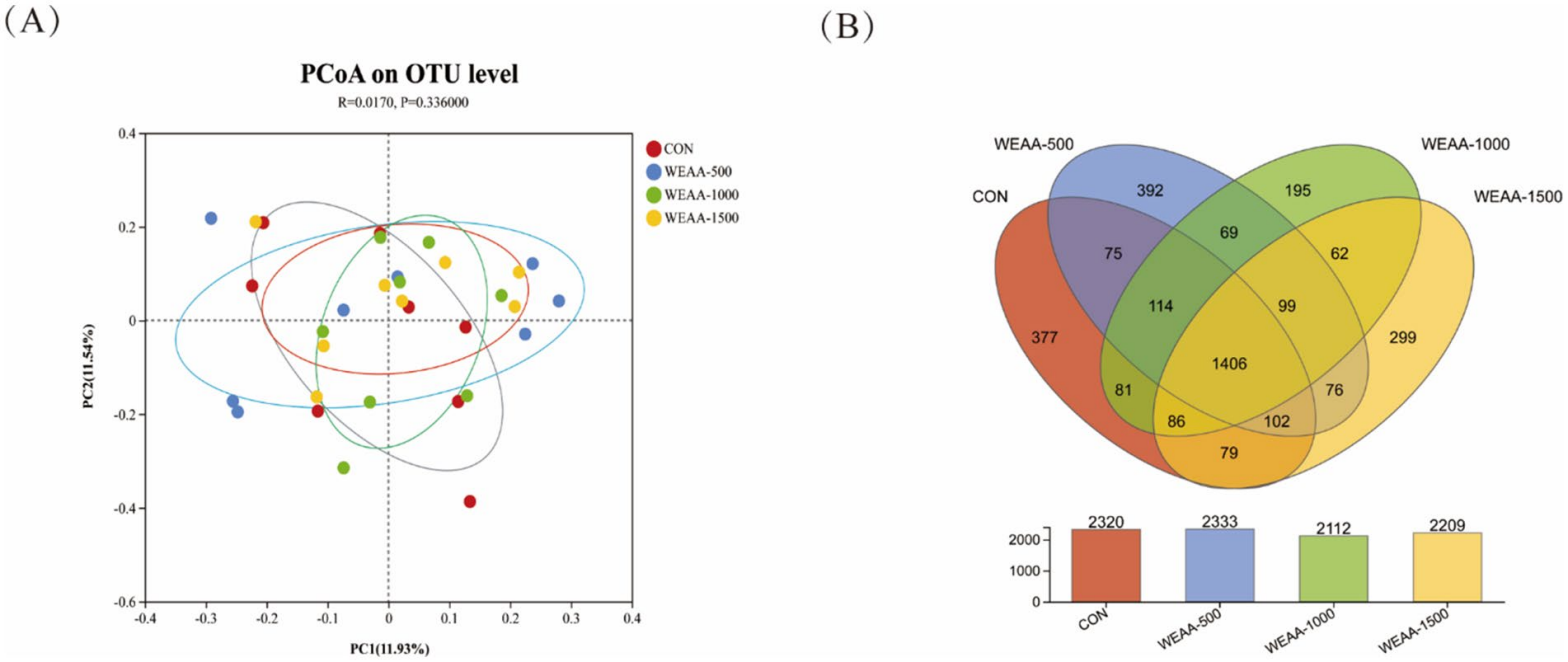
**(A)** Effects of WEAA on the expression in the rumen microbial beta diversity of lambs. **(B)** The Venn diagram of the shared and unique OTUs throughout the four different added groups. Values were expressed as the mean of 8 lambs in each group and the same below. WEAA: Water Extracts of *Artemisia annua* L. CON: control; WEAA-500: 500 mg/kg WEAA; WEAA-1000: 1000 mg/kg WEAA; WEAA-1500: 1500 mg/kg WEAA.

The rumen microbiota’s Principal Coordinate Analysis (PCoA) relying on Brey-Curtis distance matrices between each sample was shown in Figure 2A. The contribution values of principal component PC1 and PC2 accounted for, respectively, 11.93 and 11.54% of the total variation. The samples of each group were represented by distinct color points, and the distance among the samples reflected the similarities and difference in the community composition of the samples. The results showed that rumen microbiota structure was not clearly separated, and there was overlapping between the control and the WEAA-supplemented group (*p* = 0.336), and their contribution to the total variation of rumen microbial communities was 1.7% (*R* = 0.017). Each group did not show an aggregation distribution, suggesting that there was not a significant variance in rumen microbial *β* diversity between the groups (*p* > 0.05).

#### 3.4.4 Species composition analysis

##### 3.4.4.1 Veen diagram

In this sequencing analysis, various OTUs were classified based on a similarity threshold of 97%. The Venn diagram is shown in Figure 2B. The total number of OTUs was 3,512 OTUs. In the control group, the number of OTUs was 2,320, and the number of unique OTUs was 377, accounting for 10.73% of the total number of OTUs. In WEAA-500 group, the number of OTUs was 2,333, and the number of unique OTUs was 392, accounting for 11.16% of the total number of OTUs. In WEAA-1000 group, the number of OTUs was 2,112, and the number of unique OTUs was 195, accounting for 5.55% of the total number of OTUs. In WEAA-1500 group, the number of OTUs was 2,209, with 299 unique OTUs, accounting for 8.51% of the total number of OTU. 1,406 OTUs (40.03% of the total) were shared among the four groups.

##### 3.4.4.2 Rumen microbial community composition

**Figure 3:**
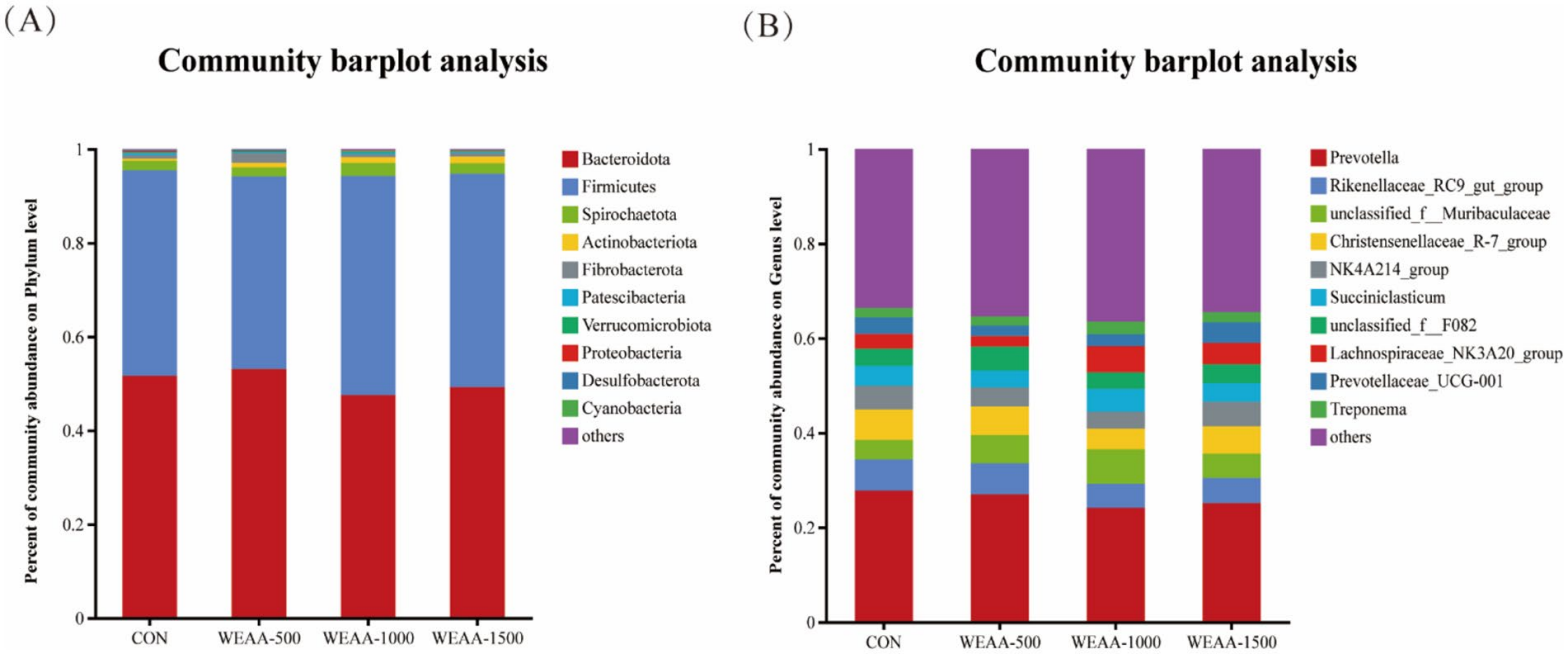
Relative abundance of ruminal microbiota at the **(A)** phylum and **(B)** genus levels. Values were expressed as the mean of 8 lambs in each group and the same below. WEAA: Water Extracts of *Artemisia annua* L. CON: control; WEAA-500: 500 mg/kg WEAA; WEAA-1000: 1000 mg/kg WEAA; WEAA-1500: 1500 mg/kg WEAA.

This study conducted a more in-depth examination of how dietary supplementation with WEAA affects rumen microbiota. In the course of this experiment, researchers identified 18 distinct microbial phyla at the phylum level, and the relative abundance (Taxa >0.1%) of the top ten species regarding ruminal microbial phyla was displayed. As shown in Table 10 and Figure 3A, the dominant taxa in the four treatment groups comprised *Bacteroidota* (50.38%), *Firmicutes* (44.26%), *Spirochaetota* (2.23%), *Actinobacteriota* (1.04%), *and Fibrobacterota* (1.02%). Notably, *Firmicutes* and *Bacteroidetes* collectively accounted for 90% of rumen microbial diversity in lambs. With the increase of WEAA supplementation, the abundance of *Actinobacteriota* exhibited a significant linear increase (*p* < 0.05), the abundance of *Cyanobacteria* showed a quadratic increasing trend (*p* < 0.10).

**Table 10:** Effects of WEAA supplementation on microbial abundance of rumen at phylum level (Top 10 phylum).

Item	WEAA mg/kg ^(1)^				SEM ^(2)^	*P*-Value ^(3)^	
	0	500	1,000	1,500		Linear	Quadratic
Bacteroidota	51.65	53.06	47.52	49.24	1.43	0.326	0.621
Firmicutes	43.80	41.07	46.72	45.49	1.47	0.422	0.706
Spirochaetota	2.01	1.96	2.75	2.20	0.28	0.595	0.795
Fibrobacterota	0.68	0.62	0.63	0.64	0.08	0.841	0.957
Actinobacteriota	0.50	0.93	1.26	1.48	0.16	0.022	0.071
Patescibacteria	0.48	0.17	0.33	0.28	0.05	0.286	0.218
Proteobacteria	0.25	0.17	0.10	0.14	0.03	0.239	0.340
Desulfobacterota	0.23	0.17	0.09	0.11	0.03	0.152	0.313
Verrucomicrobiota	0.19	0.15	0.23	0.11	0.02	0.510	0.583
Cyanobacteria	0.06	0.07	0.11	0.11	0.01	0.017	0.052
Others	0.17	0.14	0.26	0.20	0.03	0.428	0.717

(1) Values were expressed as the mean of 8 lambs in each group and the same below. WEAA: Water Extracts of *Artemisia annua* L.

(2) SEM = Standard error of the mean.

(3) Dose-dependent effects of WEAA. The probability value of *p* ≤ 0.05 was considered to be statistically significant, whereas the probability value of 0.05 < *p* < 0.10 was considered as a tendency.

At the Genus level, a total of 258 microbial Genera were identified, and the relative abundance (Taxa >0.1%) of the top ten species in terms of ruminal microbial Genus-level was presented. *Prevotella* (26.00%), *Rikenellaceae_RC9_gut_group* (5.89%), *Christensenellaceae_R-7_group* (5.63%), *norank_f_Muribaculaceae* (5.64%), and *norank_f_F082* (4.04%) were found to be dominant taxa across all four treatment groups. Table 11 and Figure 3B demonstrated that the relative abundance of *Lachnospiraceae_NK3A20 group* showed a linear increasing trend (*p* < 0.10) with increasing dietary supplementation of WEAA.

**Table 11:** Effects of WEAA dietary supplementation on microbial abundance of rumen at genus level (Top 10 Genus).

Item	WEAA mg/kg ^(1)^				SEM ^(2)^	*P*-Value ^(3)^	
	0	500	1,000	1,500		Linear	Quadratic
Prevotella	27.73	26.95	24.15	25.09	1.85	0.526	0.801
Rikenellaceae_RC9_gut_group	6.59	6.59	5.05	5.33	0.53	0.271	0.547
Unclassified_f_Muribaculaceae	4.16	5.96	7.30	5.13	0.83	0.573	0.426
Christensenellaceae_R-7_group	6.40	6.06	4.31	5.78	0.56	0.480	0.572
NK4A214_group	5.04	4.01	3.59	5.14	0.35	0.970	0.182
Succiniclasticum	4.24	3.51	4.82	4.01	0.50	0.892	0.990
Unclassified_f_F082	3.59	5.08	3.48	3.98	0.59	0.935	0.919
Lachnospiraceae_NK3A20_group	3.12	2.28	5.59	4.53	0.48	0.078	0.215
Prevotellaceae_UCG-001	3.54	1.98	2.47	4.32	0.65	0.638	0.394
Treponema	1.96	1.92	2.70	2.18	0.28	0.578	0.785
Others	33.62	35.66	36.53	34.52	1.13	0.729	0.641

(1) Values were expressed as the mean of 8 lambs in each group and the same below. WEAA: Water Extracts of *Artemisia annua* L.

(2) SEM = Standard error of the mean.

(3) Dose-dependent effects of WEAA. The probability value of *p* ≤ 0.05 was considered to be statistically significant, whereas the probability value of 0.05 < *p* < 0.10 was considered as a tendency.

##### 3.4.4.3 Multilevel species difference discrimination analysis

**Figure 4:**
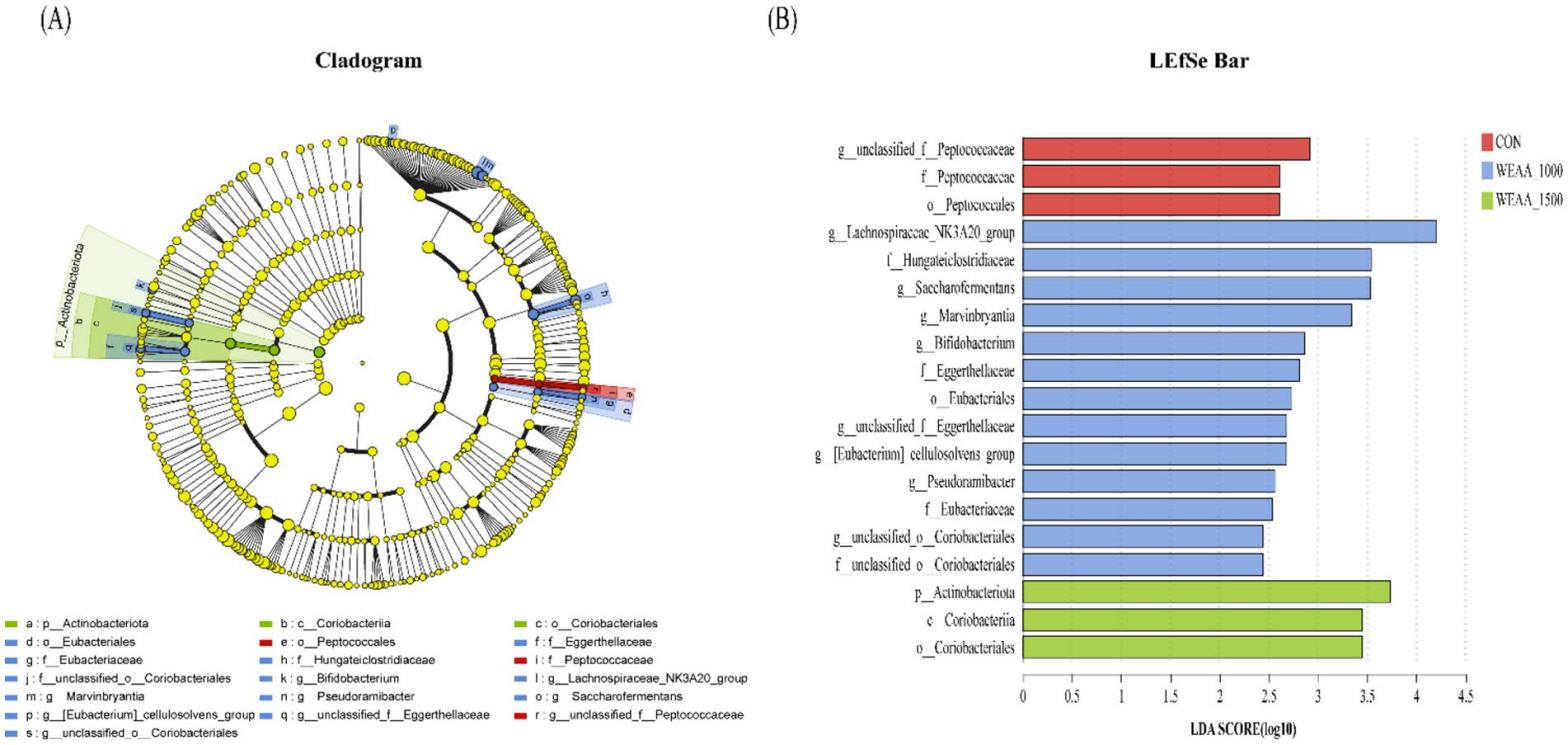
Analysis using LefSe of the ruminal microbiota across four groups with varying levels of addition. **(A)** The linear discriminant analysis (LDA) score for the rumen microbiota indicates that a score of ≥2 signifies statistical significance. **(B)** The cladogram generated by LEfSe illustrates the taxonomic profiling beginning at the genus level. The yellow node indicates no significant difference, whereas nodes in other colors highlight notable differences. Values were expressed as the mean of 8 lambs in each group and the same below. WEAA: Water Extracts of *Artemisia annua* L. CON: control; WEAA-1000: 1000 mg/kg WEAA; WEAA-1500: 1500 mg/kg WEAA.

To ascertain the functional communities within the samples, an LEfSe analysis was conducted to discern the particular microorganisms present in the rumen fluid of the four treatment groups (as depicted in Figures 4A,B). Microorganisms with a linear discriminant analysis (LDA) value greater than 2 were regarded as specific microorganisms among the four groups. A total of 19 specific microflora were obtained from 32 rumen fluid samples of lambs, in which, *g_unclassified_f_Peptococcaceae* were significantly abundant in the control group; the *g__Lachnospiraceae_NK3A20_group*, *g_Saccharofermentans*, *g_Marvinbryantia*, *g_Bifidobacterium*, *g_unclassified_f_Eggerthellaceae*, *g_[Eubacterium]_cellulosolvens_group*, *g_Pseudoramibacter*, and *g_unclassified_o_Coriobacteriales* were significantly abundant in the 1,000 mg/kg WEAA supplementation group.

#### 3.4.5 Spearman’s correlation analysis

**Figure 5:**
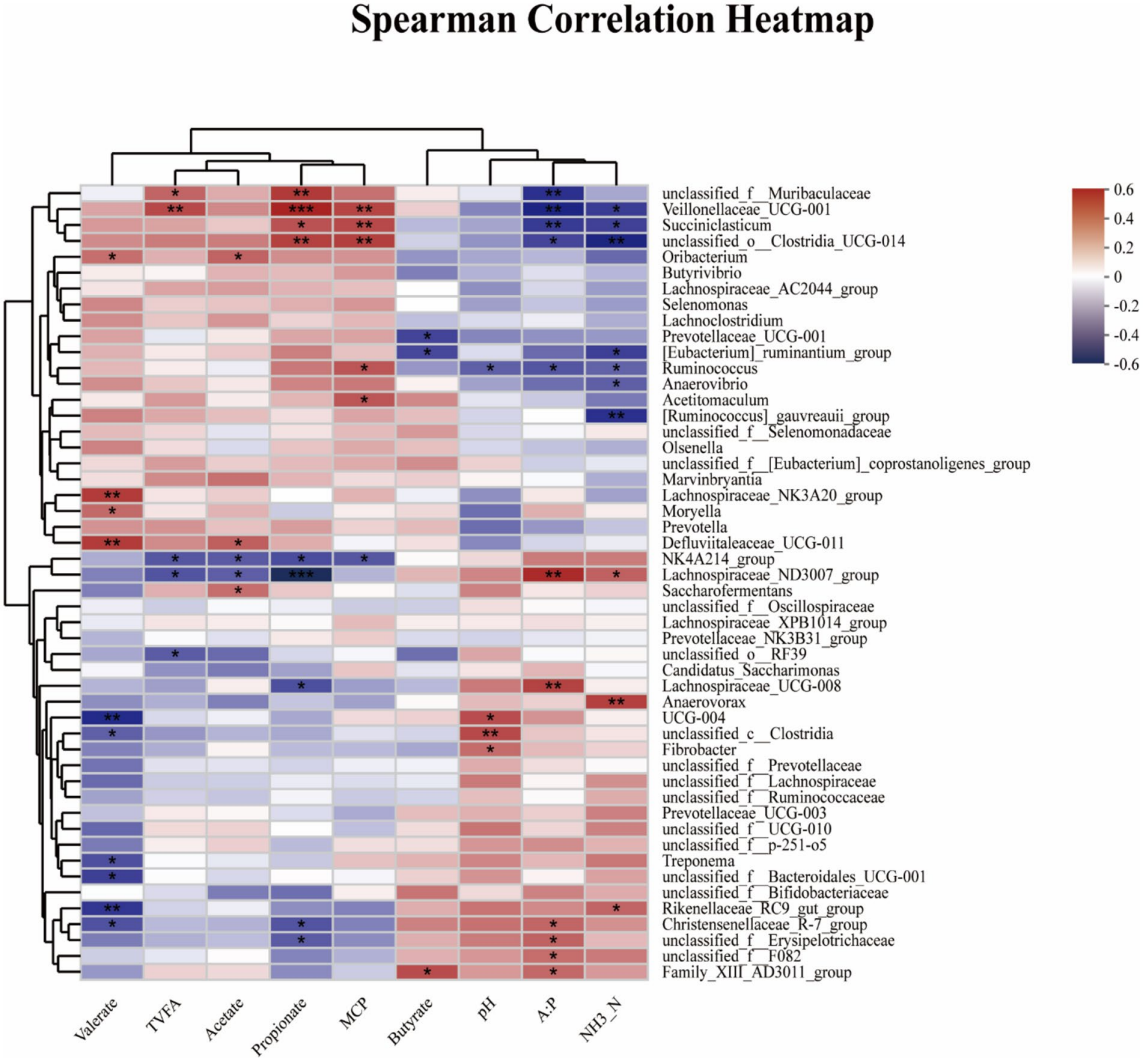
Spearman’s correlation analysis between ruminal fermentation parameters and microbial diversity and abundance. Spearman’s correlation coefficients (R) are given, with R values displayed in different colors, respectively, and the legend on the right is the color interval of different R values. *, ** and *** represent *p* < 0.05, 0.01 and 0.001, and the same applies below. TVFA = Total volatile fatty acid; A:P = Acetate/propionate ratio. pH = Potential of hydrogen; NH_3_-N = Ammonia nitrogen; MCP = Microprotein.

Figure 5 showed Spearman’s correlation analysis between ruminal fermentation parameter and any of the most abundant microbial genera (top 50 genera), in which several positive and negative correlations were observed between ruminal fermentation and rumen microbia. The pH was positively associated with the abundance of *g__UCG-004* (R = 0.4398, 0.01 < *p* < 0.05), *g_unclassified_c_Clostridia* (*R* = 0.4508, *p* < 0.01), and *g_Fibrobacter* (R = 0.3587, 0.01 < *p* < 0.05), while it showed a negative relationship with the abundance of *g__Ruminococcus* (*R* = −0.3629, 0.01 < *p* < 0.05). The concentration of NH_3_-N was positively associated with the abundance of *g_Rikenellaceae_RC9_gut_group* (*R* = 0.3757, 0.01 < *p* < 0.05), *g_Lachnospiraceae_ND3007_group* (R = 0.3867, *p* < 0.01), and *g_Anaerovorax* (*R* = 0.4777, 0.01 < *p* < 0.05), while it showed a negative relationship with the abundance of *g_unclassified_o_Clostridia_UCG-014* (*R* = −0.5132, *p* < 0.01), *g_[Ruminococcus]_gauvreauii_group* (*R* = −0.4790, *p* < 0.01), *g_Veillonellaceae_UCG-001* (*R* = −0.4480, 0.01 < *p* < 0.05), *g_Anaerovibrio* (R = −0.3662, 0.01 < *p* < 0.05), *g_[Eubacterium]_ruminantium_group* (*R* = −0.4397, 0.01 < *p* < 0.05), *g_Succiniclasticum* (*R* = −0.4363, 0.01 < *p* < 0.05), *g_Ruminococcus* (*R* = −0.3563, 0.01 < *p* < 0.05). The MCP content was positively associated with the abundance of *g_unclassified_o_Clostridia_UCG-014* (*R* = 0.4787, *p* < 0.01), *g_Veillonellaceae_UCG-001* (*R* = 0.4615, *p* < 0.01), *g_Succiniclasticum* (R = 0.4524, *p* < 0.01), *g_Ruminococcus* (*R* = 0.4150, 0.01 < *p* < 0.05), and *g_Acetitomaculum* (*R* = 0.4174, 0.01 < *p* < 0.05), while it showed a negative relationship with the abundance of *g_NK4A214_group* (*R* = −0.3820, 0.01 < *p* < 0.05). The TVFA was positively associated with the abundance of *g_Veillonellaceae_UCG-001* (*R* = 0.4509, *p* < 0.01), *g_unclassified_f_Muribaculaceae* (*R* = 0.3765, 0.01 < *p* < 0.05), while it showed a negative relationship with the abundance of *g_NK4A214_group* (*R* = −0.3820, 0.01 < *p* < 0.05), *g_Lachnospiraceae_ND3007_group* (*R* = −0.3911, 0.01 < *p* < 0.05), *g_unclassified_o_RF39* (*R* = −0.3705, 0.01 < *p* < 0.05). The concentration of acetic acid was positively associated with the abundance of *g_Saccharofermentans* (*R* = 0.3512, 0.01 < *p* < 0.05), *g_Oribacterium* (*R* = 0.3806, 0.01 < *p* < 0.05), *g_Defluviitaleaceae_UCG-011*(*R* = 0.3936, 0.01 < *p* < 0.05), while it showed a negative relationship with the abundance of *g_NK4A214_group* (*R* = −0.3750, 0.01 < *p* < 0.05), *g_Lachnospiraceae_ND3007_group* (R = −0.3662, 0.01 < *p* < 0.05). The concentration of propionic acid was positively associated with the abundance of *g_Veillonellaceae_UCG-001* (*R* = 0.5556, *p* < 0.001), *g_unclassified_f_Muribaculaceae* (R = 0.4938, *p <* 0.01), *g_unclassified_o_Clostridia_UCG-014* (*R* = 0.4740, *p* < 0.01), and *g_Succiniclasticum* (*R* = 0.4271, 0.01 < *p* < 0.05), while it showed a negative relationship with the abundance of *g_NK4A214_group* (*R* = −0.4064, 0.01 < *p* < 0.05), *g_Lachnospiraceae_ND3007_group* (*R* = −0.5860, *p* < 0.001), *g_Christensenellaceae_R-7_group* (*R* = −0.3892, 0.01 < *p* < 0.05), *g_unclassified_f_Erysipelotrichaceae* (*R* = −0.3727, 0.01 < *p* < 0.05), and *g_Lachnospiraceae_UCG-008* (*R* = −0.4005, 0.01 < *p* < 0.05). The concentration of butyric acid was positively associated with the abundance of *g_Family_XII_AD3011_group* (*R* = 0.4393, 0.01 < *p* < 0.05), while it showed a negative relationship with the abundance of *g_[Eubacterium]_ruminantium_group* (*R* = −0.4205, 0.01 < *p* < 0.05), *g_Prevotellaceae_UCG-001* (*R* = −0.4260, 0.01 < *p* < 0.05). The concentration of valeric acid was positively associated with the abundance of *g_Oribacterium* (*R* = 0.3495, 0.01 < *p* < 0.05), *g_Defluviitaleaceae_UCG-011* (R = 0.4913, *p* < 0.01), *g_Lachnospiraceae_NK3A20_group* (*R* = 0.4922, *p* < 0.01), and *g_Moryella* (R = 0.3629, 0.01 < *p* < 0.05), while it showed a negative relationship with the abundance of *g_Christensenellaceae_R-7_group* (*R* = −0.3950, 0.01 < *p* < 0.05), *g_Rikenellaceae_RC9_gut_group* (*R* = −0.4623, 0.01 < *p* < 0.05), *g_Treponema* (R = −0.4024, 0.01 < *p* < 0.05), *g_unclassified_c_Clostridia* (R = −0.3631, 0.01 < *p* < 0.05), *g_unclassified_f_Bacteroidales_UCG-001* (*R* = −0.4384, 0.01 < *p* < 0.05), and *g_UCG-004* (*R* = −0.4901, *p* < 0.01). The A:P was positively associated with the abundance of *g_Christensenellaceae_R-7_group* (*R* = 0.3735, 0.01 < *p* < 0.05), *g_Family_XIII_AD3011_group* (*R* = 0.3683, 0.01 < *p* < 0.05), *g_Lachnospiraceae_ND3007_group* (*R* = 0.5319, *p* < 0.01), *g_unclassified_f_Erysipelotrichaceae* (*R* = 0.3820, 0.01 < *p* < 0.05), *g_Lachnospiraceae_UCG-008* (R = 0.4695, *p* < 0.01), and *g_unclassified_f_F082* (*R* = 0.3556, 0.01 < *p* < 0.05), while it showed a negative relationship with the abundance of *g_Veillonellaceae_UCG-001* (*R* = −0.5110, *p* < 0.01), *g_unclassified_f_Muribaculaceae* (*R* = −0.4762, *p* < 0.01), *g_unclassified_o_Clostridia_UCG-014* (*R* = −0.4245, 0.01 < *p* < 0.05), *g_Succiniclasticum* (*R* = −0.4517, *p* < 0.01), and *g_Ruminococcus* (*R* = −0.3878, 0.01 < *p* < 0.05).

**Figure 6:**
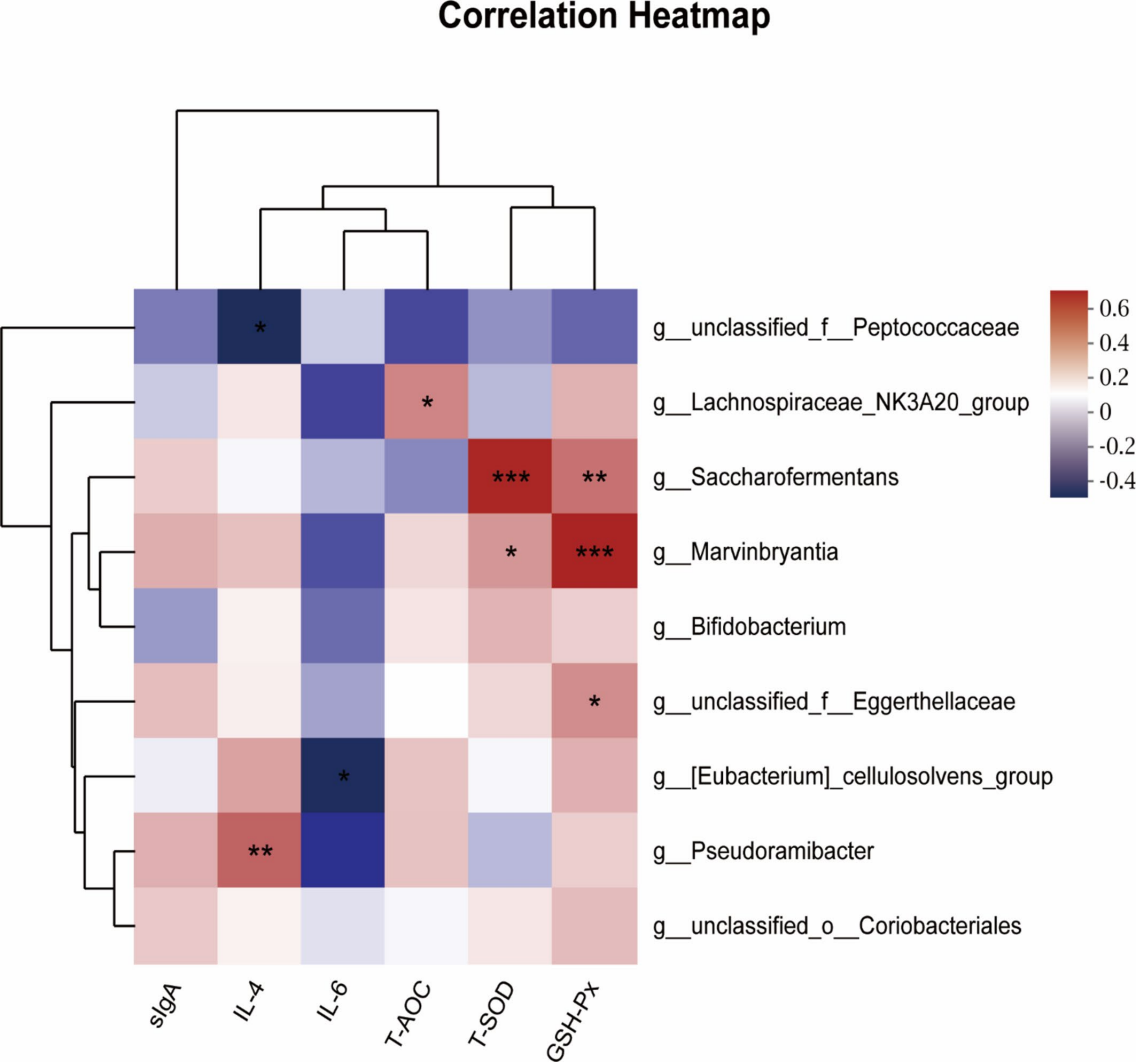
Spearman’s correlation analysis between rumen antioxidant and inflammatory status, and ruminal microbiota by LefSe analysis of ruminal microbiota from genus level. Spearman’s correlation coefficients (R) are given, with R values displayed in different colors, respectively, and the legend on the right is the color interval of different R values. *, ** and *** represent *p* < 0.05, 0.01 and 0.001. sIgA = Secretory immunoglobulin A; IL-4 = Interleukin-4; IL-6 = Interleukin-6; T-AOC = Total antioxidant capacity; T-SOD = Total superoxide dismutase; GSH-Px = Glutathione peroxidase.

Figure 6 presented the Spearman’s correlation analysis regarding the relationship between rumen inflammation and antioxidant levels, as well as the ruminal microbiota, through LefSe analysis of the ruminal microbiota at the genus level. Here, several positive and negative correlations were identified between the rumen inflammation and antioxidant levels and the ruminal microbiota. The IL-4 was positively associated with the abundance of *g_Pseudoramibacter* (*R* = 0.4943, 0.01 < *p* < 0.05), while it was negatively correlated with the abundance of *g_unclassified_f_Peptococcaceae* (*R* = −0.4249, *p* < 0.05). The IL-6 was found to be negatively associated with the abundance of *g_[Eubacterium]_cellulosolvens_group* (*R* = −0.4135, *p* < 0.05). T-AOC exhibited a positive relationship with the abundance of *g_Lachnospiraceae_NK3A20_group* (*R* = 0.4044, *p* < 0.05). T-SOD was positively associated with the abundance of *g_Saccharofermentans* (R = 0.6504, *p* < 0.001), and *g_Marvinbryantia* (*R* = 0.3614, *p* < 0.05). GSH-Px was positively associated with the abundance of *g_Saccharofermentans* (*R* = 0.4553, *p* < 0.01), *g_Marvinbryantia* (*R* = 0.6576, *p* < 0.001), and *g_unclassified_f_Eggerthellaceae* (*R* = 0.3890, *p* < 0.05).

## 4 Discussion

### 4.1 Rumen immune and antioxidant indexes and relevant gene expression

Type 1 helper (Th1) and Type 2 helper (Th2) cells exert a vital role in the inflammatory response by secreting a diverse array of cytokines, including IL-1β, IL-2, IL-4, IL-6, and TNF-*α*. The body’s normal immune function can be maintained by modulating Th1/ Th2 balance (Crome et al., 2010). sIgA is the primary immunoglobulin within the gastrointestinal mucosa, actively contributing to both humoral and cellular immunity processes within the body (Keiichiro and Akira, 2014). The present study revealed that the supplementation of WEAA led to an increase in the rumen contents of sIgA, IL-2, IL-4, as well as the gene expression of IL-4. This finding is comparable to the previous study where it was suggested that the dietary *Artemisia annua* L. water extract could boost the IgM and sIgA content in the jejunum and ileum of broilers (Guo et al., 2022). Additionally, feeding WEAA groups reduced the gene expression of iNOS and COX-2 in the rumen tissue, which suggested that WEAA could boost immune activity in rumen through suppressing gene expression. Our findings were consistent with those of Zhang et al. (2020), who reported that dietary supplementation with *Artemisia argyi* extract significantly suppressed the serum level of NO and iNOS and the gene expression of iNOS in broilers challenged by LPS. Furthermore, it was regulated by several signaling pathways associated with inflammation, particularly the TLR4/NF-κB pathway. In our study, supplementing WEAA groups increased the expression of MyD88 in the rumen tissue, but had no significant effects on IKKβ, NFκBp50, NFκBp65, IL-1β, and TNF-α gene expression. It is well known that *Artemisia annua* L. is rich in flavonoids (Song et al., 2017). It has been demonstrated that flavonoids of plant origin have anti-inflammatory effects (Zhao et al., 2023, 2024), which is related to the suppression of the NF-κB signal transduction pathway and the reduction of IκB-α phosphorylation (Zeinali et al., 2017; Vezza et al., 2016; Romier et al., 2008). Notably, in our study, WEAA significantly up-regulated the gene expression level of IκB by 23–47%, indicating that the immune-modulatory and anti-inflammatory attributes of the extracts could potentially be modulated by means of an elevation in the IκB to suppress the NF-κB signaling pathway, and this phenomenon can elucidate the reduction in the expression of iNOS and COX-2.

In the breeding of sheep, many factors such as intensive breeding, feeding conditions, feeding density and changes in normal behavior habits can impact the organism’s metabolic processes and immune defense mechanisms, consequently influencing production performance (Tao et al., 2020). Some studies have shown a positive correlation between the antioxidant capacity and the presence of polyphenols or flavonoids in natural herbs, with these compounds being identified as the primary components responsible for antioxidant activity (Qin et al., 2017; Škerget et al., 2005). In addition, antioxidants and their corresponding enzymes play a crucial role in the maintenance of cellular homeostasis. For instance, T-AOC, T-SOD, GSH-Px, CAT are able to shield the body from oxidative stress damage, bolster the elimination of reactive oxygen species (ROS) (Niu et al., 2018; Huchzermeyer et al., 2022). By contrast, MDA is one of the trustworthy markers utilized for the evaluation of oxidative stress (Giera et al., 2012). In the present study, compared with the control group, the supplementing WEAA groups characterized by a relatively lower transcription of MDA. *Artemisia annua* L. contains phenolic compounds and flavonoids which may increase antioxidant enzyme activity (Brisibe et al., 2009; Gouveia and Castilho, 2013). Payam et al. (2019) when they fed laying hens with *Artemisia annua* leaves, it positively affected the plasma antioxidant status. Specifically, the concentration of GSH-Px was raised, and the MDA was lowered. Ryu et al. (1998) feeding rats with Artemisia extracts increased liver GSH-Px and decreased MDA production. In addition, *Artemisia annua* leaves and enzymatically treated *Artemisia annua* could in varying degrees increase antioxidant enzyme activities (T-AOC, T-SOD, GSH-Px, CAT) and decrease MDA production in the serum and liver in broilers (Wan et al., 2016). These results align with the findings of the present study, which showed that WEAA supplementation increased the level of T-SOD, GSH-Px and the gene expression of *SOD2*, *GSH-Px*, and *HO-1* in rumen tissue. It is well known that under normal physiological status or low lipid peroxidation rate, the organism itself has the ability to regulate cellular metabolic circumstances through constitutive antioxidant defense systems or signaling pathways activation, which up-regulate antioxidants enzyme activity resulting in an adaptive stress response (Ahmad et al., 2022). In general, SOD helps superoxide (O^2−^) convert into H_2_O_2_ and uses relative signaling pathways to activate the alternative scavenging enzymes (SOD1, CAT, GSH-Px, HO-1) and detoxify H_2_O_2_ into H_2_O (Wu et al., 2013). The above antioxidant mechanism may be regulated by nuclear translocation of Nrf2 into the nucleus. Xing et al. (2021) indicated that *Artemisia ordosica* polysaccharide partially mitigated oxidative stress in broilers via the Keap1/ Nrf2 pathway. Consequently, we ascertained the mRNA expression levels of *Nrf2* and *Keap1*, and found that WEAA supplementation groups notably enhanced the expression of *Nrf2* and reduced the expression of *Keap1* in the rumen tissue. Hence, WEAA is a green feed additive with a potential to improve rumen immunomodulatory and antioxidant properties in lambs.

### 4.2 Rumen fermentation

The fermentation parameters of rumen can reflect the homeostasis of the rumen environment, and even the efficiency of energy and protein utilization to a certain extent. The pH level is a direct indicator of the health of the ruminal environment, which is closely related to dietary composition and additives. The optimal range of ruminal fluid pH (between 6.2 and 7.2) can modulate VFA-producing pathways and is beneficial to the absorption of VFAs (Van and Peter, 2019; Dijkstra et al., 2012). Besides, Briggs et al. (1957) observed a negative correlation between VFA concentrations and pH. In the present experiment, the pH of rumen fluid ranged from 6.7 to 7.2 and remained unaffected by WEAA treatment. Although a lower pH was observed in WEAA-fed animals, no statistically significant difference was detected, indicating that WEAA had no impact on the acid–base equilibrium in the rumen. Some studies have shown that the production of volatile fatty acids (VFA) in the rumen increases, leading to a decrease in pH value when plant essential oils are added to high-quality feed for cattle (Castillejos et al., 2008). This explains the fluctuation of rumen fluid pH in this study. In common with the present study, no effect on pH was reported with the addition of 25 and 50% *Artemisia sieberi* leaves (Faryabi et al., 2023). NH₃-N constitutes a crucial element in the rumen of ruminant animals for the synthesis of microbial protein. It functions as the principal nitrogen source, facilitating the production of essential proteins by microbes. The presence of NH₃-N is of paramount significance for the optimal operation of the rumen microbial ecosystem and the overall well-being and productivity of ruminants. The normal concentration range is 5–30 mg/dL (Grummer et al., 1984). Furthermore, Chen et al. (2021) observed a decreasing trend in rumen NH_3_-N concentrations in lambs fed a diet containing probiotics and Chinese medicine polysaccharide compounds, with all groups maintaining NH_3_-N concentrations within the range. The findings of our study are similar with these results. The WEAA supplementation decreased the concentration of NH_3_-N in rumen fluid of lambs significantly. Furthermore, the addition of WEAA led to a significant increase in the concentration of microbial protein (MCP), suggesting that WEAA promoted rumen MCP synthesis using ammonia nitrogen (Jiang et al., 2020). Our study found that supplementing WEAA in the diet of lambs increased the concentrations of TVFA, propionate, while also reduced the A:P in the rumen fluid. In addition, *Veillonellaceae_UCG-001* and *unclassified_o_Clostridia_UCG-014* belong to the phylum *Firmicutes*, and *Veillonellaceae_UCG-001* contains many cellulose-decomposing bacteria (Chen et al., 2022; Niu et al., 2022; Jiang et al., 2023). *Succiniclasticum*, a gram-negative rod-shaped anaerobe, is capable of fermenting succinate and converting it to propionate, an essential precursor of glucose in the rumen (Van Gylswyk, 1995; McLoughlin et al., 2023). The results of Spearman’s correlation analysis found that *Veillonellaceae_UCG-001* positively correlated with TVFA, propionate, and MCP concentration, *unclassified_o_Clostridia_UCG-014* and *Succiniclasticum* positively correlated with propionate and MCP concentration, as well as *Veillonellaceae_UCG-001*, *unclassified_o_Clostridia_UCG-014* and *Succiniclasticum had* a significant negative correlation with the NH_3_-N and A:P. This was consistent with the results of our fermentation parameter. Similarly, Yu et al. (2021) supplemented varying doses of *Artemisia annua* extract (0, 0.25, 0.50, 0.75%) in the diets of lactating cattle, and found a significant increase in concentration of TVFA, propionic acid, and butyric acid in rumen of the *Artemisia annua* extract group, along with a notable decrease in the ratio of acetic acid to propionic acid, and *Artemisia annua* extract supplementation also improved the immunity and antioxidant properties of dairy cows. Furthermore, Faryabi et al. (2023) investigated the impacts of including 25% *Artemisia sieberi* leaves in the diet on the ruminal fermentation of growing male lambs. It was demonstrated that *Artemisia sieberi* leaves resulted in a considerable rise in propionate concentrations and an enhancement in the blood antioxidant level. In light of the above-mentioned discoveries, it could be inferred that WEAA affected the rumen microbial composition, and these microorganisms were able to bring about a substantial decrease in the A:*p* value by employing cellulose and hemicellulose as substrates to generate VFA and increased propionate concentration, and to enhance MCP synthesis using ammonia nitrogen, and augmented the immune and antioxidant functions by its influence on the levels of VFAs and increased the absorption rate of VFAs in the rumen epithelium.

### 4.3 Rumen microbiota diversity

The rumen is a highly diverse, complex, and stable ecosystem, consisting of a variety of microorganisms (bacteria, protozoa, archaea, and fungi) that have different abundance and diversity within the host organism (Denman et al., 2018). The rumen microbial domains are essential to the viability of the host. Through prolonged natural selection and evolution, the rumen microbes and host establish an interactive and mutually dependent homeostatic relationship that significantly contributes to maintaining host health, enhancing performance (Zeineldin et al., 2018; Qu et al., 2023; Zhang et al., 2022; Zhang et al., 2022). The present experimentation demonstrated that dietary addition of WEAA did not affect the Ace, Chao1, Shannon, and Simpson indices of the rumen microbial community. Also, there was no substantial dissimilarity in rumen microbial *β* diversity among groups, suggesting that the WEAA had no adverse effects on rumen microbial richness and diversity of the rumen microbial community in lambs, and could maintain microbial homeostasis in the rumen.

The microbiota composition, and several significantly distinct genera in the 1,000 mg/kg WEAA addition group and CON groups were found to differ in this study. A large number of studies showed that the dominant phyla in ruminant rumen were *Firmicutes* and *Bacteroidetes*, which accounted for about 95% of the total microbial population (Harry, 1997), and our study found that the total number of *Firmicutes* and *Bacteroidetes* in each experimental groups both account for 95% of the total microorganisms.

The addition of WEAA with a value of 1,000 mg/kg in the present study increased the relative abundance of *Actinobacteriota* and three genera in this phylum, including *Bifidobacterium*. *Actinobacteria* are one of the major organic matter-decomposing microbes. Numerous studies have shown that *Actinobacteria* are probiotics with the ability to degrade polysaccharides. These *Actinobacteria* can convert feedstuffs into microbial biomass and fermentation end products that can be utilized by the animal host, thereby contributing to the host’s health (Dhanasekaran and Jiang, 2016). However, *Bifidobacterium*, a beneficial genus in this phylum, is an important microbial group in the mammalian gastrointestinal tract that helps to improve digestive problems, enhance immunity, and exhibit antioxidant activity (Lin et al., 2021). In the 1,000 mg/kg WEAA addition group, there were several significantly distinct genera, all belonging to *Firmicutes* (*g__Lachnospiraceae_NK3A20_group*, *g__Saccharofermentans*, *g__Marvinbryantia*, and *g_ [Eubacterium] _cellulosolvens_group*). Previous research demonstrated that colitis in mice could be relieved through oral administration of microbiological inosine, which was achieved by increasing the abundance of beneficial bacteria (*g_Lachnospiraceae_NK3A20_group*, *g_Romboutsia*, *g__Marvinbryantia*, and *g_Bifidobacterium*) (Guo et al., 2023). The *g_Lachnospiraceae_NK3A20_group*, a member of the family *Lachnospiraceae*, is estimated to constitute 2.7% of rumen bacteria based on 16S rRNA gene surveys of rumen microbiota (Kaminsky et al., 2023). Some studies have demonstrated that members of the *Lachnospiraceae* family can degrade polysaccharides, produce butyric acid, and enhance antioxidant activity (Henderson et al., 2015; Biddle et al., 2013). The results of the LEfSe analysis in this study indicate that the *g__Lachnospiraceae_NK3A20_group* is positively correlated with T-AOC activity in the rumen. The *Saccharofermentans* genus exhibits the capacity for the degradation of cellulose and hemicellulose, resulting in substantial propionate production (Tian et al., 2021; Rettenmaier et al., 2021), thus providing an explanation for the significant increase in propionic acid and reduction in A:*p* values. Additionally, *Marvinbryantia* has been identified as potentially playing a role in gastrointestinal tract health-related issues in animals (Lawson and Rainey, 2015), which can contribute to improving fermentation and the production of acetate as its primary metabolic product (Rey et al., 2010). It was noteworthy that the results of the present study were further supported by LEfSe analysis, indicating a significant positive correlation between *Saccharofermentans* and T-SOD activity, as well as a positive correlation with GSH-Px in rumen tissue. The Marvinbryantia genus demonstrated a considerable positive association with the activities of GSH-Px and T-SOD. Furthermore, the *g_ [Eubacterium]_cellulosolvens_group* shows a negative connection with IL-6 concentration. Taken together, this research speculated that WEAA might contribute to the maintenance of rumen health by modulating the abundance of dominant bacteria, thereby intervening internal environmental dysregulation of rumen microorganisms and altering their metabolites to activate immune and antioxidant pathways.

## 5 Conclusion

The inclusion of WEAA in the diet could regulate the rumen immune response and antioxidant activity via the NF-κB and Nrf2 signaling pathway. Furthermore, WEAA enhances rumen fermentation in lambs. This phenomenon may be explained by the increased diversity and altered structure of the rumen microflora. As a result of WEAA supplementation, the colonization of beneficial bacteria has improved, including *Actinobacteriota*, *Cyanobacteria* and *Lachnospiraceae_NK3A20_group*. In addition, *g__Lachnospiraceae_NK3A20_group, g_Saccharofermentans*, *g_Marvinbryantia*, *g_Bifidobacterium* were significantly abundant as specific microflora in the 1,000 mg/kg WEAA supplementation group. The optimal dosage of WEAA in the diet of lambs was determined to be 1,000 mg/kg diet.

## Data Availability

The original contributions presented in the study are publicly available. This data can be found here: https://www.ncbi.nlm.nih.gov/, accession number PRJNA1168294.
